# Partitioning Evapotranspiration into Green and Blue Water Sources in the Conterminous United States

**DOI:** 10.1038/s41598-017-06359-w

**Published:** 2017-07-21

**Authors:** Naga Manohar Velpuri, Gabriel B. Senay

**Affiliations:** 1 ASRC InuTeq LLC, Contractor to the US Geological Survey (USGS) Earth Resources Observation and Science (EROS) Center, Sioux Falls, SD USA; 2 US Geological Survey (USGS), Earth Resources Observation and Science (EROS) Center, North Central Climate Science Center, Fort Collins, CO USA

## Abstract

In this study, we combined two 1 km actual evapotranspiration datasets (ET), one obtained from a root zone water balance model and another from an energy balance model, to partition annual ET into green (rainfall-based) and blue (surface water/groundwater) sources. Time series maps of green water ET (GWET) and blue water ET (BWET) are produced for the conterminous United States (CONUS) over 2001–2015. Our results indicate that average green and blue water for all land cover types in CONUS accounts for nearly 70% and 30% of the total ET, respectively. The ET in the eastern US arises mostly from GWET, and in the western US, it is mostly BWET. Analysis of the BWET in the 16 irrigated areas in CONUS revealed interesting results. While the magnitude of the BWET gradually showed a decline from west to east, the increase in coefficient of variation from west to east confirmed greater use of supplemental irrigation in the central and eastern US. We also established relationships between different hydro-climatology zones and their blue water requirements. This study provides insights on the relative contributions and the spatiotemporal dynamics of GWET and BWET, which could lead to improved water resources management.

## Introduction

The concept of green and blue water is relatively new^1^. When precipitation reaches land, it takes either green or blue water pathways^2^. The water that is stored in the unsaturated soil layer forms the green water resource and the water that is stored in the rivers, streams, surface-water bodies and groundwater forms the blue water resources. Problems such as water scarcity and water security are changing the way we understand, use, and manage green and blue water resources for food production and ecosystems. One of the recommendations to alleviate water scarcity is to reduce consumptive water use in the agriculture sector^3^. However, understanding water use in crop production by source (rain water or irrigation water from surface and groundwater) is vital for water resource management^4^. Information on how much of direct rain water (green water) and how much of non-rain water (blue water) in the form of irrigation is being productively used is critical for efficient management of water resources. However, such information is not readily available.

By definition, crop or vegetation evapotranspiration (ET) comes from green water sources (i.e., from the water consumed by the vegetation from the root zone soil moisture and soil evaporation from the unsaturated soil surface). However, when vegetation falls short of green water sources, they are often supplied with or have the ability to extract blue water resources (such as irrigation from rivers, reservoirs, or groundwater). While doing so, blue water is converted to green water before it is lost as ET. However, in terms of analysis of source of ET, we considered the water lost from a blue water source as a blue water ET. Hence, we define the total ET (*ET*_*Total*_) as

1 $$E{T}_{Total}=GWET+BWET$$

where *GWET* and *BWET* are the green and blue water ET sources, respectively. Definitions and equations to compute actual green and blue water ET are explained in depth in the water footprint assessment manual^5^.

Conventional approaches to estimate the BWET use a water balance model or a combination of models such as H08 model^6^, global crop water model^7^, GEPIC model^8^, all of which use data on climate, soil, crop characteristics, national statistics, reports, crop-related maps, and actual irrigation as input^5^. However, running hydrologic models over very large areas becomes data intensive and time-consuming. Nevertheless, these models have been used to produce global maps of the blue water (or green water) ET for croplands only^6–9^. Furthermore, the quantity of the outputs depend strongly on the assumed growing areas and cropping seasons of the specific crop type and how well the irrigated and rainfed crop production is distinguishable^7^. Overcoming these limitations and estimating and understanding the GWET and BWET across all landscapes is important for efficient water resources management.

In this study, we developed a simple but robust methodology to partition ET into GWET and BWET. Theoretically, water balance ET (WBET) produced from our VegET water balance model^10^ is a spatially explicit (1 km grid cell), one-dimensional root-zone water balance model that is driven by precipitation, operating on a control volume defined by the root zone (1 m deep) using a key parameter- water holding capacity (WHC) to define the size of the “bucket” and hence captures ET only from green water sources. Furthermore, the parameters used in this model are not influenced by the land surface processes (meteorological parameters produced from atmospheric models), WBET represents true natural conditions and thereby captures green water use only. On the other hand, energy balance ET (EBET) implicitly takes into account the impact of water stress (rainfall, irrigation, or groundwater) regardless of the source of moisture; hence, it captures the sum of green ET and blue ET. Because of their modeling approaches, both WBET and EBET estimates are often not directly comparable. But they should agree on rainfed areas where the water in natural ecosystem is available only from green water sources. However, due to differences in input data and modeling approach, estimates of ET from both WBET and EBET often fail to agree even on natural landscapes due to presence of model error. One solution to this problem is to validate and bias correct ET datasets using a reliable reference dataset. In this study, we used a gridded FLUXNET ET dataset^11, 12^ obtained from the Max Planck Institute (MPI-ET) dataset for validation and bias correction of WBET and EBET datasets. We use bias-corrected WBET and EBET datasets to partition GWET and BWET. This methodology is simple in its physical principles but robust in the way we produce ET datasets. Our method is similar to the approach used in a recent study^13^, which used the bias-correction approach to correct two actual ET products such as Global Land Data Assimilation System-based actual ET that represents green water use and remote sensing-based actual ET that represents a combination of GWET and BWET. In this study we used a root zone water balance model that provides high resolution GWET estimates and bias-corrected ET datasets that can provide GWET and BWET for all landscapes.

The objective of this study is to develop an approach to partition ET into green and blue water sources. The specific objectives of this study are to i) understand spatial and temporal dynamics of GWET and BWET, ii) analyze sources of ET for different land cover types, and iii) quantify GWET and BWET of irrigated croplands across the conterminous United States (CONUS). Such information on the relative contributions of GWET and BWET in irrigated agriculture will help improve our understanding of blue water use for irrigation.

## Results

### Validation and bias correction of ET datasets

Two ET datasets, water balance ET obtained from the VegET root zone water balance model^10, 14^ and energy balance ET obtained from the operational simplified surface energy balance model (SSEBop) ET dataset^15^, were validated using the Max Planck Institute ET (MPI ET) dataset. MPI ET has been cross-validated against eddy covariance measurements and independent ET measurements, and was found to explain more than 90% of variance^12^. Since then, the dataset has been used as a proxy for *in situ* measurements of ET for validating model ET estimates^16, 17^. Compared to MPI ET data, both EBET and WBET showed high correlation with mean R^2^ of 0.89 and 0.79, respectively (Table 1). However, EBET showed higher mean root mean square error (RMSE) than WBET (129 and 73 mm/year, respectively). Similarly, mean bias error (MBE) was also found to be higher for EBET than WBET with mean estimates of 69 and 24 mm/year, respectively (Table 1).Validation scatterplots pre- and post-bias correction are provided in the Supplementary information.

**Table 1:** Validation and bias correction of energy balance ET (EBET) and water balance ET (WBET) annual datasets using the MPI ET dataset.

Year	Coefficient of Determination (R^2^)		Pre-Bias Correction				Post-Bias Correction			
			RMSE (mm/year)		MBE (mm/year)		RMSE (mm/year)		MBE (mm/year)	
	EBET	WBET	EBET	WBET	EBET	WBET	EBET_BC_	WBET_BC_	EBET_BC_	WBET_BC_
2001	0.91	0.79	136	69	92	22	100	66	0	0
2002	0.92	0.89	138	60	67	19	121	56	0	0
2003	0.91	0.85	126	61	73	14	102	58	0	0
2004	0.95	0.91	144	62	86	7	115	61	0	0
2005	0.90	0.70	131	87	79	36	104	81	0	0
2006	0.87	0.66	135	97	95	65	96	71	0	0
2007	0.94	0.89	154	55	54	31	144	83	0	0
2008	0.88	0.71	127	91	74	24	104	89	0	0
2009	0.88	0.85	114	71	74	10	87	71	0	0
2010	0.88	0.78	128	77	25	6	126	77	0	0
2011	0.77	0.64	81	74	45	34	67	71	0	0
Mean	0.89	0.79	129	73	69	24	106	71	0	0

Subscript ‘BC’ indicates bias corrected dataset. Please note bias correction has been performed on the subset of the grassland HUCs (on HUCs with >90% grassland cover).

Next, we corrected both the ET datasets for bias error obtained during the validation process. After bias correction, the RMSE was reduced up to 18% and 3% in the bias-corrected EBET and WBET datasets, respectively. After bias correction, the bias error in the datasets was totally eliminated (Table 1).

### Validation of bias corrected ET datasets

**Figure 1:**
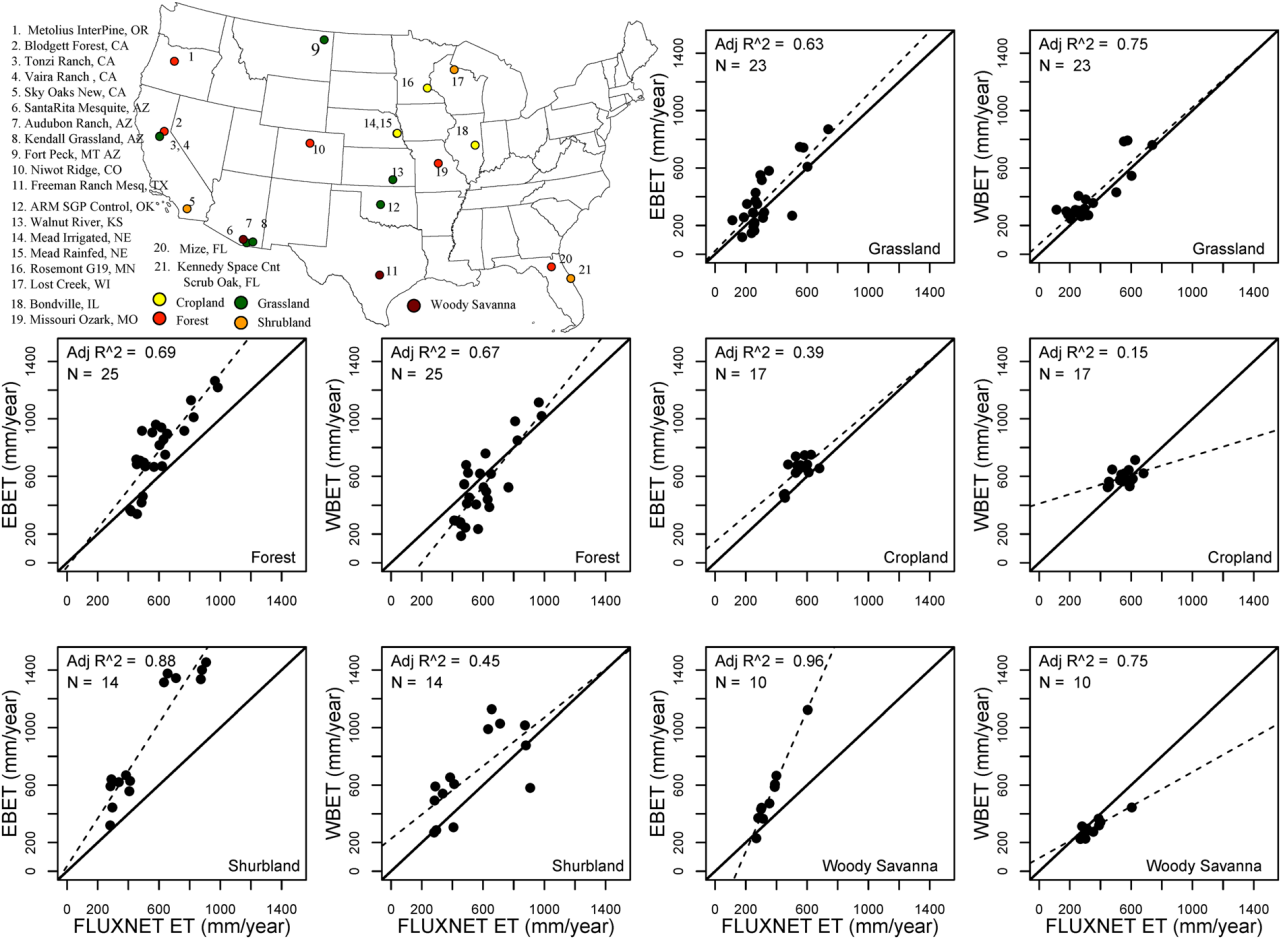
Validation of bias corrected EBET and WBET datasets using data collected over 2001–2007 from 21 FLUXNET sites grouped into five distinct land cover types. The figure was generated using R 3.3.0 software https://www.r-project.org/.

Validation results (Fig. 1) reveal a good correspondence of bias corrected ET estimates with *in situ* flux tower data. The bias corrected ET obtained from energy balance (EBET) and water balance (WBET) showed a good fit with almost zero bias for grassland class indicating the effectiveness of the bias correction procedure. Although cropland ET from both EBET and WBET matches well with *in situ* data, lack of a marked range (values clustered around mean) in ET estimates resulted in lower R^2^. When compared to EBET, WBET estimates were of lower magnitudes (less than *in situ* ET) for woody savanna and forest classes. This is mainly because the water balance model only captures ET from precipitation (green ET). On the other hand, EBET estimates show better agreement (higher R^2^) with *in situ* data for most classes and are higher than the WBET estimates as expected. This is because they captured both natural and anthropogenic effects (blue and green ET). Overall, the adjusted R^2^ estimates for most classes was found to be reasonable for a national scale study.

### Green water and blue water ET maps

**Figure 2:**
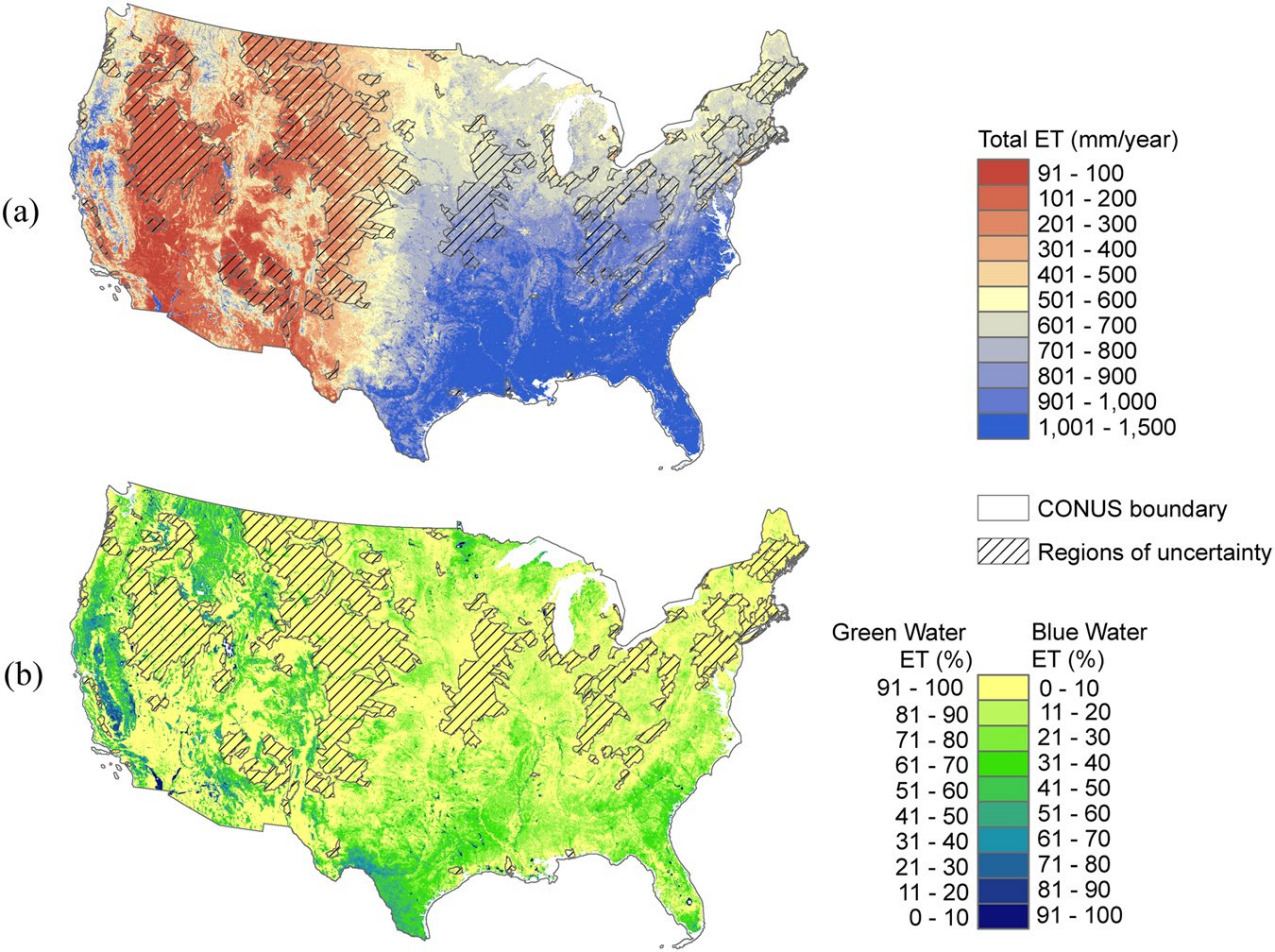
Long-term (2001–2015) average estimates of (**a**) annual evapotranspiration (ET), (**b**) GWET and BWET contributions (%) to the total ET for the conterminous United States. The figure was generated using ArcGIS (ArcMap 10.3.1) software http://desktop.arcgis.com/en/arcmap/.

Annual GWET and BWET maps were produced for 2001–2015. Average estimates of annual total ET (mm/year), percentage of annual GWET, and BWET maps are shown in Fig. 2. As expected, total annual ET is higher in the east and southeastern United States and gradually reduces from east to west (Fig. 2a). However, areas of agriculture and some forested landscapes in the west also show high total ET. It was estimated that average annual GWET and BWET for all land cover classes in CONUS account for nearly 70% and 30% of the annual ET, respectively.

Figure 2b indicates the relative contribution of the GWET and BWET to total ET. In the eastern United States, the dominant water source of ET arises from green water. As expected, the GWET dominated in the arid to semiarid regions of the west where precipitation is the only source of water. Only the agricultural areas of the west and some forested landscapes in the west to northwestern United States show a lower GWET. The BWET map is the inverse of the GWET map. Most regions in the east to northeast show a very low (<10%) BWET, as precipitation meets most of the water demand. However, some regions in the southern United States have a BWET of >50%. Except for a few areas, most of the central and north-central United States also shows a low BWET. In the west, except for a few patches of high BWET, most of the area shows a very low (<10%) BWET. For example, the open shrubland areas in the west survive mostly on green water resources (precipitation). The high BWET in the west comes mostly from the regions where water from non-rainfall sources contributes to the vegetation growth. For example, the agricultural (irrigated) areas in the Central Valley in California and the regions covered by surface-water bodies. It is interesting to see that large areas of the Sierra Nevada Mountain ranges and evergreen forests of the northwest contribute to high BWET. This could possibly be attributed to the ability of these landscapes (such as forests in the northwest and grassland/mesquite habitats in the south) to tap shallow to deeper groundwater resources.

### Green and blue water ET analysis for different land cover types

**Figure 3:**
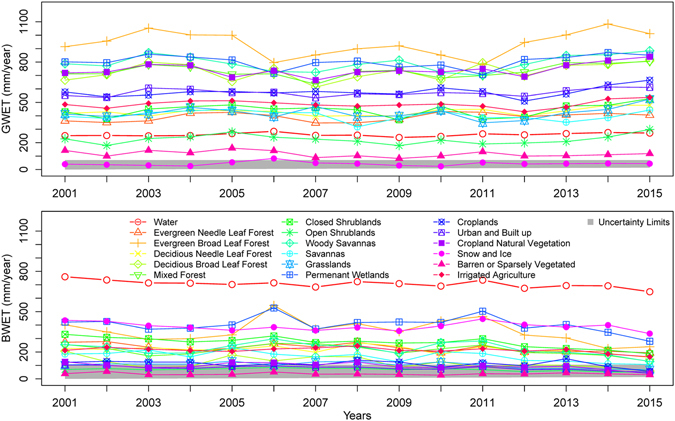
Average annual green water ET (GWET) and blue water ET (BWET) summarized for different land cover types. The gray polygon indicates the region where mean estimates of the BWET and GWET are within the range of modeling error/uncertainty (derived using post-bias correction RMSE). The figure was generated using R 3.3.0 software https://www.r-project.org/.

The Moderate Resolution Imaging Spectroradiometer (MODIS) 500-m land cover climatology product^18^ was used to understand GWET and BWET of different land cover types. Summaries of GWET and BWET for 18 land cover types are presented in Fig. 3. Results indicate that evergreen broadleaf forests, woody savannas, and permanent wetland regions show the highest GWET with estimates in excess of 700 mm/year. On the other hand, only ET over surface-water bodies show the highest (>700 mm/year) BWET. Obviously, the land cover types that have unlimited water sources (such as snow and ice, and permanent wetlands) show a greater BWET than other land cover types. The BWET for most of the other land cover types is less than 500 mm/year, and the evergreen broadleaf forest class shows the highest blue water use (~350 mm/year) among non-water classes.

The croplands class showed GWET and BWET of about 87% and 13%, respectively, of total ET. This cropland class includes both irrigated and rainfed croplands where water sources vary substantially. Hence, summaries of GWET and BWET exclusively from irrigated areas are also presented. To do so, we used an irrigated area map for CONUS derived by merging MODIS and national agricultural statistics obtained from the U.S. Geological Survey^19^. Countrywide analysis of the irrigated croplands class showed GWET and BWET of about 69% and 31%, respectively, of total ET. Again, the GWET is still higher than the BWET as this irrigated class also includes large areas of supplemental irrigation where the green water use is dominant. The GWET and BWET of 18 selected classes did not show any statistically significant trend over 2001–2015. Based on the error in the post-bias correction datasets (RMSE in Table 1), we suggest that land cover types with mean ET estimates falling within the error limits of the model should be analyzed with caution.

### Dynamics of blue water ET of selected irrigated landscapes

**Figure 4:**
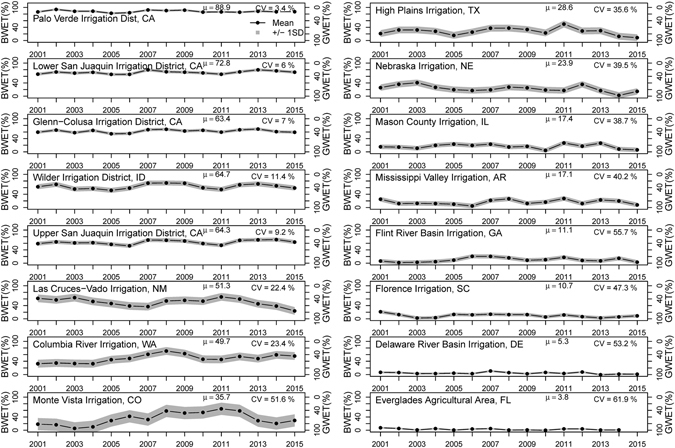
Time series plots (2001–2015) of average annual blue and green water ET (BWET and GWET) summarized for 16 irrigated areas in CONUS. The gray area denotes ±1 standard deviation. μ and CV indicates the mean and coefficient of variation for BWET. The figure was generated using R 3.3.0 software https://www.r-project.org/.

We analyzed the BWET over 16 different irrigated areas distributed across CONUS (Fig. 4). Magnitude and variability of mean ± 1 standard deviation of the annual GWET and BWET (in percent) over 2001–2015 are presented in Fig. 4. Across all 16 irrigated areas, the BWET showed high variability (from ~90% to <5% of total annual ET). The Palo Verde Irrigation District (PVID) located in California consistently showed a high (89 ± 3%) BWET across all years, whereas the Everglades Agricultural Area (EAA) located in Florida was found to consistently show the lowest (4 ± 2%) BWET. Even though irrigated areas in the central United States showed the highest standard deviation, the coefficient of variation of BWET increased from west to east. This could be explained by the use of blue water resources (as supplemental irrigation). The range and temporal dynamics were found to be negligible in the east and the west; however, irrigated areas in the central United States showed higher temporal dynamics. For example, Monte Vista Irrigation in Colorado showed the highest standard deviation with a BWET of 36 ± 18%. This is mostly because of the unreliability of precipitation at critical stages of crop growth when farmers depend on blue water resources.

Moreover, the average GWET and BWET in Fig. 4 shows a clear west to east gradient in magnitude. We found that irrigated areas located in the semiarid to arid regions of the western US require more blue water (hence higher BWET) than in the eastern US. In the eastern United States, however, crop water demand was found to be met mostly by green water sources. The central United States was found to show a varying requirement of green and blue water depending on the annual variability in precipitation.

**Figure 5:**
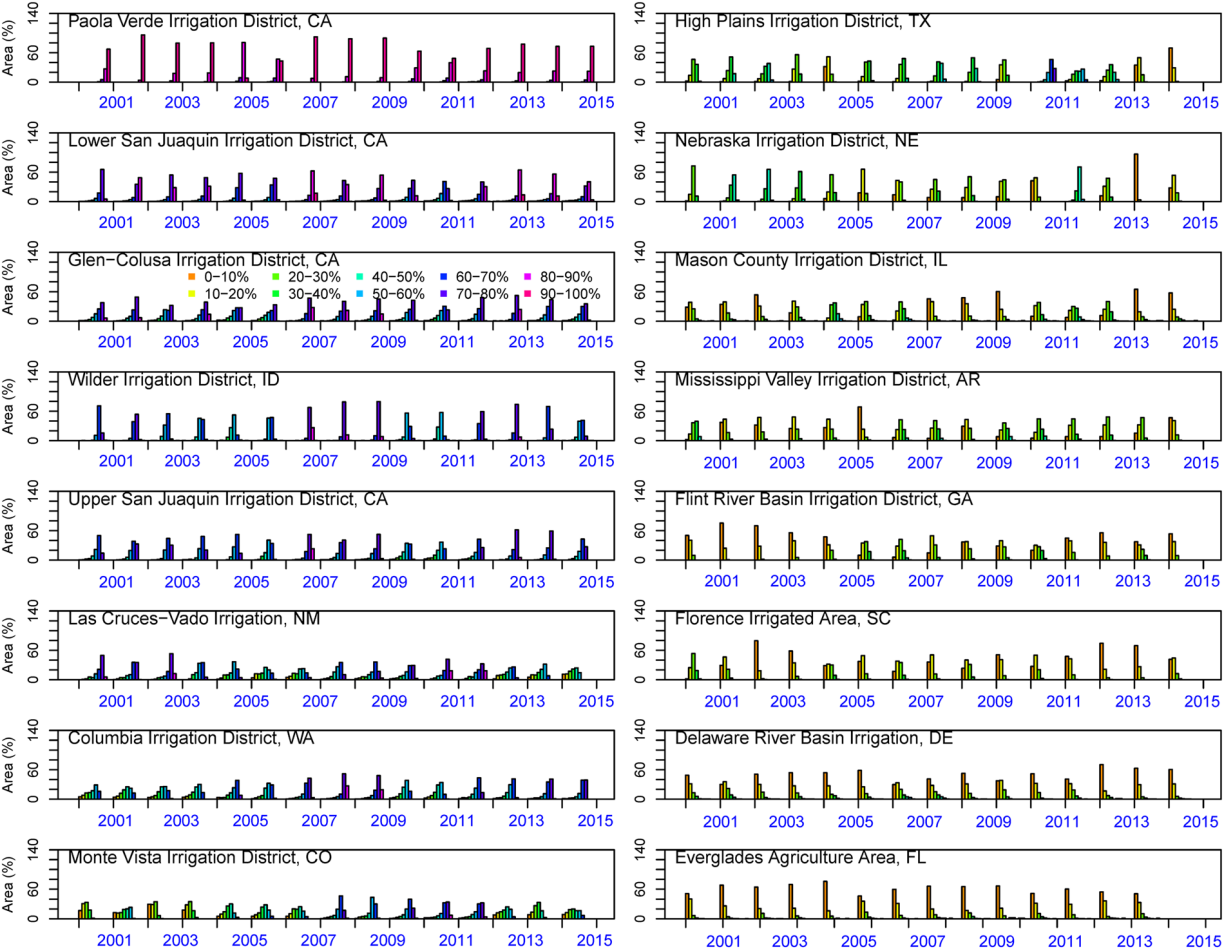
Spatial variability in the BWET: frequency distribution of irrigated landscapes with different categories of BWET percent summarized for 16 different irrigated areas across CONUS. The figure was generated using R 3.3.0 software https://www.r-project.org/.

There is usually high spatial variability in the GWET or BWET within any given irrigation district. Differences in crop type, ownership, irrigation scheduling, and water availability based on the location cause this variability. Although Fig. 4 provides magnitude and temporal variability in the BWET, we cannot determine how much the BWET varies spatially within a given irrigated area. To further understand the spatial variability of the BWET within each irrigated area, we plotted histograms showing percent area under different categories of BWET within an irrigation district. Figure 5 shows that histograms tend to shift from areas under high BWET to areas under low BWET from west to east (from magenta to yellow-orange).

In general, increasing atmospheric demand during dry years can lead to increase in the BWET. However, we argue that water management decisions are more responsible for the interannual variability in the blue water use. For example, in PVID, we do not see a significant increase in BWET in spite of prolonged drought in California. This is mainly because of the fallowing program in place in the PVID that reduces the extraction of blue water from Lake Mead, which is used for irrigation. This provides additional water to the metropolitan water district of the greater Los Angeles area. Similarly, other irrigation districts such as irrigated areas in Nebraska and the High Plain Irrigation District in Texas are showing declines in blue water use. This decline could be attributed to the reduction in groundwater pumped from the Ogallala High Plains aquifer following passage and implementation of Farm Bill – 2008. Since then, the Natural Resources Conservation Service (NRCS), a Federal agency has overseen reductions in groundwater use up to 1.5 million acre feet (https://www.nrcs.usda.gov/Internet/FSE_DOCUMENTS/stelprdb1186440.pdf).

### Impact of hydro-climatology on green and blue water ET requirements

**Figure 6:**
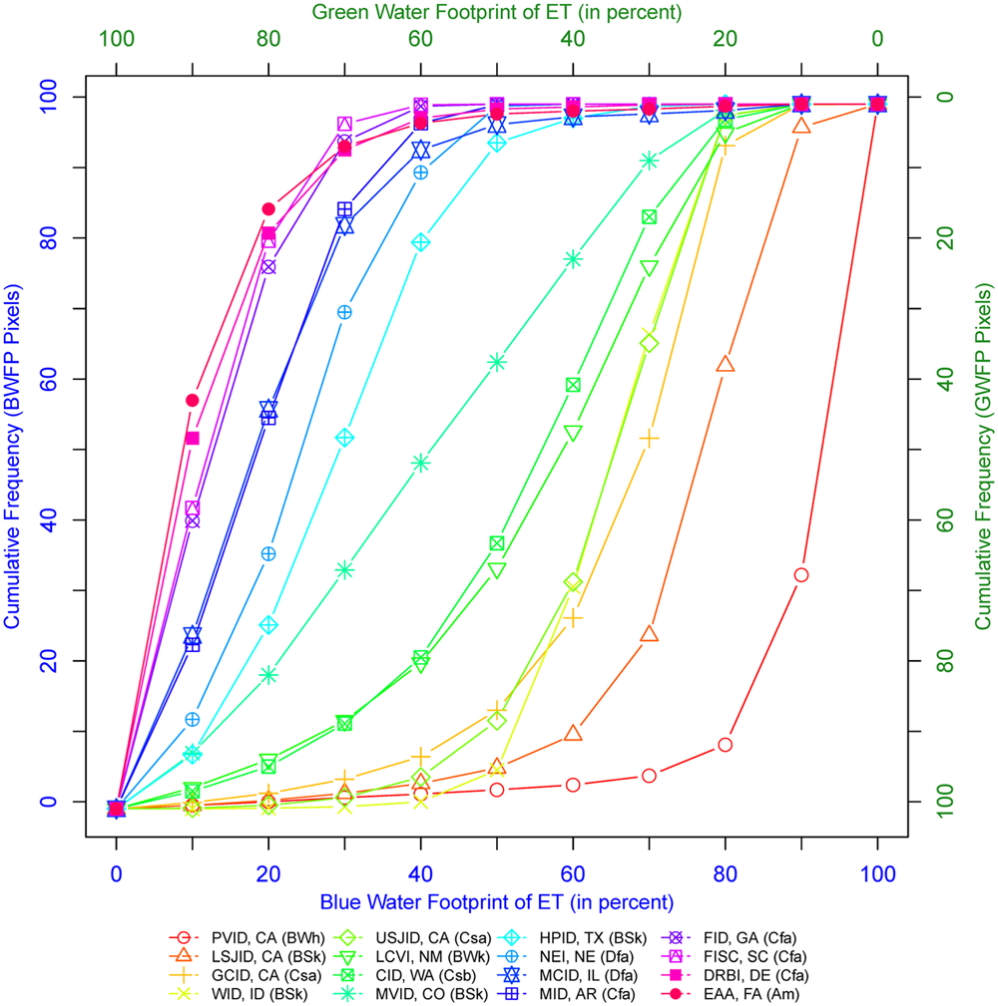
Cumulative distribution of the number of pixels with percent BWET and GWET summarized for 16 different irrigated areas and their hydro-climatic zones. The figure was generated using R 3.3.0 software https://www.r-project.org/.

We hypothesize that the hydro-climatology of the region is the reason for the west-east gradient in magnitude of GWET and BWET observed over CONUS. To test this hypothesis, we extracted hydro-climatology classes from the updated Koppen-Geiger Climatology map^20^ and plotted the cumulative frequency of pixels against its water source for each of the 16 irrigated areas in Fig. 6. The following observations can be inferred from Fig. 6. i) As expected, the cumulative frequency of ET source curves for the irrigated areas belonging to distinct hydro-climate groups occupies discrete areas in the plot. For example, irrigated areas from dry arid hydro-climates showed high BWET with an exponential-type curve, whereas irrigation districts belonging to temperate and humid hydro-climates show low BWET with a logarithmic-type curve. ii) The dominant source of ET for each irrigation district/hydro-climate can be identified using the region of steepest slope. For example, BWET is dominant (>80%) and is coming from nearly 90% of the area in the Palo Verde Irrigation District (located in a dry, arid hydro-climate). iii) It is possible to identify the dominant ET source for a given irrigation district when its hydro-climatology is known. For example, nearly 30% of the pixels (or area) within an irrigation district located in the arid to semiarid regions will require 60–100% of its water to come from blue water resources. Similarly, a cropland located in the central United States will require 20–50% of its water to come from blue water resources.Detailed classification of selected irrigated areas into different hydro-climatic zones is provided in the supplimentary information.

### Errors and uncertainty in GWET and BWET estimates

Errors in the BWET and GWET estimates arise from (a) the model input data, (b) the bias-correction approach, and (c) the partitioning approach. In most physically based models, when using satellite data such as EBET and WBET, major errors in the model outputs can be attributed to the model parameters or input data. For example, the basin-scale error in the EBET dataset produced from the SSEBop ET model could range up to 25%^16^. Similarly, errors in the WBET could be up to 15–30%^21^. Furthermore, the accuracy of the validation data (MPI ET) is also important. MPI ET is the most consistent and reliable global gridded actual ET time-series dataset (1984–2011) currently available to us. However, we acknowledge that the accuracy of MPI ET could be spatially variable depending on the availability of input training datasets in the upscaling model^12^. In this study, we estimated bias using grassland-dominant hydrologic unit code 8 (HUC8) watersheds. The accuracy of MODIS land cover climatology classification can also contribute to some uncertainty in the BWET. However, due to spatial averaging over vast areas of natural grasslands (>90% area), the impact of errors of MODIS land cover climatology is minimized. Furthermore, some of the systematic component of errors in these datasets are eliminated by the bias correction. For simplicity, we estimated mean bias over all the selected HUCs and used in the bias correction. It is important to know that the bias correction approach used in this study only reduces the error by eliminating bias component of the overall error from each pixel. Validation results using independent *in situ* flux tower site data revealed reasonable correlation across major land cover types. We quantified RMSE in the bias-corrected GWET and BWET as 107 and 71 mm/year, respectively. Hence, any annual estimate of GWET and BWET within the limits of error should be analyzed with caution. Some of the error in the GWET and BWET datasets can also be attributed to the partitioning approach. Since both energy balance and water balance approaches are independent datasets, often it results in areas where BWET can be negative (where WBET >EBET). We have ignored these negative estimates in our analysis of land cover and highlighted those regions in Fig. 2 as uncertain regions. It is to be noted that since 16 irrigated areas fall outside the regions of uncertainty, the results presented for irrigated regions are not affected by these errors. Also, as pointed out by Romaguera *et al*.^13^, bias-correction impact on ET from irrigated classes is minimal^13^. However, further research is need and will be focused on improving bias estimates and reducing errors/uncertainty in the blue and green water ET estimates. Similarly, underestimation of GWET can also lead to overestimation of BWET. For example, large areas of the Sierra Nevada Mountain ranges and evergreen forests of the northwest show high BWET. Although it is possible that some forested landscapes are capable of extracting shallow to deeper groundwater, we also acknowledge that it could be due to underestimation of GWET.

## Discussion

The need for green and blue water management has been highlighted by several researchers^2, 22, 23^. Until now, research has been more focused on blue water (irrigation management/improvement, water productivity) and less on understanding the relative contributions of GWET and BWET sources^22^. The methodology presented in this study is used to partition ET into GWET and BWET over the period from 2001–2015, allowing us to improve our understanding of the relative contribution of ET sources across space and time.

At the national scale, the GWET was found to be dominant with the mean ET source for all land cover classes over CONUS attributed to nearly 70% green water and 30% blue water. The estimates of BWET could seem high as it includes surface-water bodies and wetland vegetation classes. However, for croplands only in the CONUS region, we determined that average GWET and BWET estimates amount to 87% and 13%, respectively, which is comparable to estimates from other studies^3, 6–8^.

Most of the land cover types showed some degree of dependence on blue water sources. The GWET for two classes (barren/sparsely vegetated and snow/ice) was found to be low and within the limits of the model error. Seven out of 14 non-water land cover types showed dependence on blue water resources in excess of 200 mm/year. Figure 3 also highlights the higher dependence of certain land cover types on blue water resources (surface-water bodies, return flow, or groundwater resources). For example, among all the land cover classes analyzed, the blue water requirement for evergreen broadleaf forests of coastal Florida was found to be highest; 72% of its water requirement is met by green water sources (precipitation), and the remaining 27% comes from blue water sources. To understand whether these broadleaf forests are tapping shallow groundwater resources in the region is a topic of further research. There is a scientific consensus that water for irrigation is being withdrawn from blue water sources at unsustainable rates and groundwater levels are declining^24, 25^. Extraction of blue water resources beyond the rate of recharge could not only poses a threat to agriculture and food production, but could also adversely impact other vegetation types (such as evergreen broadleaf forests) that are dependent on blue water resources (Fig. 3).

The magnitude of the BWET shown in Fig. 4 indicates that irrigated agriculture in the southwest and the west shows high dependence on blue water resources. Currently, blue water resources such as water diverted from the Colorado River (Lake Mead) and the groundwater resources in the west are critical for sustaining crop/food production and the economy. Any changes to these blue water resources could adversely affect the region. Reducing water levels in Lake Mead, which supplies water to over 5 million hectares of agriculture, is a grave concern^26^. Furthermore, prolonged drought in the region and subsequent groundwater depletion will pose a serious threat to the blue water supply in the west^27^.

Figure 4 demonstrates that irrigated areas in the central United States show the highest standard deviation in a year-to-year basis. However, the coefficient of variation was found to increase from west to east. The higher variability in the BWET toward the east means that farmers switch to blue water resources whenever there is a precipitation deficit, particularly at critical stages of crop growth. Since this depends on the crop type and the distribution of rainfall locally, there is high spatial and temporal variability in blue water use in these regions. This erratic nature (high variability) of the BWET also means that it becomes challenging for water managers to account for irrigation water use every year. The data and methods presented in this approach would provide guidance to water resource managers in the central and eastern US with efficient planning, accounting, and management of water use/supply for agriculture.

The analysis of BWET and GWET over selected irrigated regions provides several new insights into the variability and source of ET at 1 km spatial resolution. Such analysis would not have been possible using other existing products of blue water ET due to their coarse resolution data and products. For example, the median size of the irrigated area analyzed in this study (~1200 km^2^ or 140,000 ha) is much smaller than the ~12000 km^2^ pixel size (1 degree resolution) of previous products^6, 8^. Even the best resolution (~10 km) of blue water data obtained from previous studies^7, 9^ would be coarse to understand spatial variability in BWET in some of the small irrigated regions analyzed in this study. The high resolution data and results obtained from this study is highly relevant for local decision making in land and water management.

The methodology and data products generated in this study provide useful tools for improving our understanding of green and blue water requirements of vegetation. While understanding the dynamics of total ET, it is important to distinguish between different sources of blue water (surface water in rivers, streams or lakes, or groundwater) that contribute to ET. The current modeling approach does not provide such a distinction in BWET. Improved data from future satellite missions such as Gravity Recovery And Climate Experiment (GRACE) Follow-On and satellite altimetry missions such as Surface Water Ocean Topography (SWOT) would provide reliable information on changes in groundwater and surface-water storages, respectively. Future research will focus on incorporating such data and improving the model for partitioning ET into different sources of BWET.

In most areas in the US, water use statistics are generated using water withdrawal data. However, about 40% of water withdrawals in agriculture typically return to local rivers or aquifers to be available for reuse^23, 28^. One of the challenges of estimating actual water use by irrigation using withdrawal data is that different crops use water at different rates in a given location, and the same crop uses water differently in a different climatic setting within the same basin^29^. Hence in most cases, the use of water withdrawals for water use estimation provides a bloated image. By using the approach presented in this study, it would be possible to understand the relative contribution of green and blue water use for agriculture.

## Methodology

### Study area and data used

**Figure 7:**
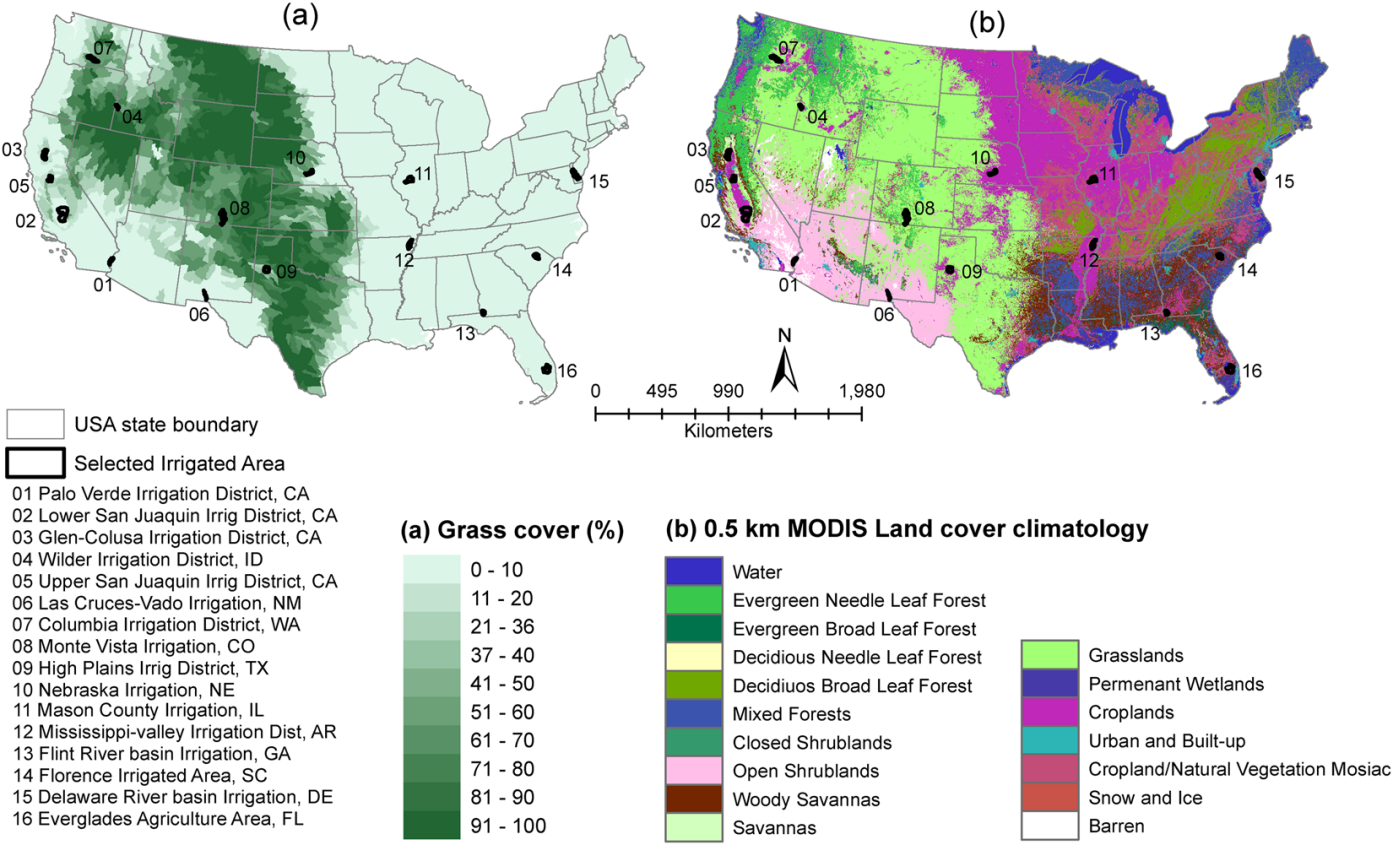
Study area showing (**a**) hydrologic units (HUC-8) dominated by grassland cover and (**b**) map of land cover climatology (0.5 km). Black areas on the map denote the locations of 16 irrigated areas analyzed in this study. The figure was generated using ArcGIS (ArcMap 10.3.1) software http://desktop.arcgis.com/en/arcmap/.

This study is restricted to the CONUS region (Fig. 7) and is primarily driven by two ET datasets. First, the gridded 1-km annual (2001–2015) water balance ET was obtained from the phenology-based VegET modeling approach^10, 21, 30^. The VegET model is a spatially explicit (1 km grid cell), one-dimensional root-zone water balance model that is driven by precipitation, operating on a control volume defined by the root zone (1 m deep) using a key parameter—water holding capacity (WHC)—to define the size of the “bucket”. The unique aspect of the VegET model is the use of remotely sensed land surface phenology to parameterize the spatial and temporal dynamics of ET on a grid-cell basis. Second, we used gridded annual 1-km energy balance ET data (2001–2015) modeled using the operational simplified surface energy balance (SSEBop) model that uses model-assimilated weather datasets and MODIS thermal images^15^ obtained from the USGS Geo Data Portal website (http://cida.usgs.gov/gdp/). Gridded 50-km annual MPI ET data (2001–2011) obtained from the Max Planck Institute were used to validate and bias correct both ET datasets. The MPI ET data were compiled using a machine-learning approach for the upscaling of eddy covariance measurements, meteorological data, climate data, and the fraction of absorbed photosynthetic active radiation data^11, 12^. Seventeen land cover classes following the International Geosphere-Biosphere Programme (IGBP) classification system^31^ obtained from the 0.5-km MODIS land cover climatology dataset^18^ (https://landcover.usgs.gov/global_climatology.php) were used to summarize green and blue water sources of ET. We used an irrigated area map for the CONUS region obtained by merging MODIS and national agricultural statistics obtained from the USGS^19^ (https://earlywarning.usgs.gov/USirrigation) and hydrologic units (HUC-8 sub-basins) boundary data obtained from the USGS (http://water.usgs.gov/GIS/huc.html). To summarize green and blue water requirements over irrigated areas, we arbitrarily selected and digitized the boundaries of 16 irrigated areas distributed across the United States. We used an updated 50-km Koppen–Geiger climate classification^20^ (http://koeppen-geiger.vu-wien.ac.at/) based on temperature and precipitation from 1951 to 2000 to classify irrigated areas into different hydro-climatic zones.

### Validation and bias correction of ET datasets using MPI ET

The main objective of performing validation is to estimate error indices such as coefficient of determination (R^2^), mean bias error (MBE), and root mean square error (RMSE), and identify the systematic bias in the datasets. The two ET models used in this study have entirely different physical model parameterizations (energy balance vs. water balance). Hence they approach ET differently. The water balance models focus on tracking pathways and magnitude of rainfall in the soil-vegetation system, i.e., it estimates ET only as a result of rainfall processes because that is the major model driver thereby capturing only rainfall based (green) ET. On the other hand, energy balance models use land surface temperature to partition radiant energy at surface into heat and ET fluxes regardless of source types rainfall, irrigation, groundwater etc). Therefore, energy balance models capture both rainfall-based (green) and non-rainfall based (blue) ET. Because of the way they are modeled, only ET estimates from natural landscapes can be compared between ﻿the﻿ two models. This is because rainfall (green water) is the only source for ET in the natural landscapes. Thus, the natural landscape such as grasslands represent areas where the two models are expected to agree.

First, we selected HUC-8 polygons that have more than 90% grassland cover (dark green polygons in Fig. 7). A high threshold for grassland cover (>90% grassland cover) is used to capture only the ET signal from rainfed areas. There are several studies that indicate strong correlation between grassland biomass with precipitation^32–34^. Estimates of ET were then extracted from the two input and reference datasets. Error indices were then estimated to understand the uncertainty in the input datasets. Estimates were checked for outliers (contamination) and only the HUCs with a pure signal of ET coming from rainfed, natural ecosystems were used. For example, several grasslands have been invaded partially or completely by mesquites in the Rio Grande Basin. These mesquites have a deep root system^35^ with the ability to tap groundwater. Such HUCs that do not represent a true rainfed, natural ecosystem were excluded from bias correction. We identified and removed HUCs based on a standard outlier detection technique using residuals. Finally, a bias error for each year for each dataset was estimated using MPI ET data (2001–2011) as

2 $$bia{s}_{i,wbet}=\frac{1}{N}\sum _{i}^{N}(MPIE{T}_{i}-WBE{T}_{i})\,{\rm{and}}\,bia{s}_{i,ebet}=\frac{1}{N}\sum _{i}^{N}(MPIE{T}_{i}-EBE{T}_{i})$$

where *i* is the year, N is the number of grassland HUCs and *bias* is the bias error for each year. Using bias error for each year, we corrected both WBET and EBET datasets using the following equations.

3 $$WBE{T}_{BC,i}=\,WBE{T}_{i}\pm bia{s}_{i,wbet}$$

4 $$EBE{T}_{BC,i}=EBE{T}_{i}\pm \,bia{s}_{i,ebet}$$

where *BC* is the bias-corrected dataset. We applied mean bias for the years when bias estimates were not available (2012–2015).

### Validation of bias corrected ET using FLUXNET data

We used level 4 latent heat flux data^36^ from 21 eddy covariance FLUXNET sites (79 station-years) for the years 2001–2007 (see Fig. 7) obtained from the Oak Ridge National Laboratory’s AmeriFlux website (http://ameriflux.ornl.gov/). FLUXNET is a global network of micrometeorological flux measurement sites that measure the exchange of CO_2_, water vapor and energy between the biosphere and the atmosphere^36, 37^. The tower-measured monthly latent heat flux (LE, W/m^2^) data were converted to ET (mm/month) using the proportionality parameter between energy and depth units of ET^38^ as

5 $$ET=\,\frac{LE}{\lambda }$$

where λ is the latent heat of vaporization (2.45 MJ/kg); LE is comparable energy units of MJ/m^2^/day and ET in mm/day (i.e., 1 MJ/m^2^/day = 0.408 mm/day with a water density of 1000 kg/m^3^). Finally, annual ET estimates (mm/year) for each station were computed and used to validate the bias corrected annual EBET and WBET estimates. Any station with missing monthly data in a given year was ignored during annual computation.

### Approach to partition ET source

Green and blue water ETs for each year (*GWET* and *BWET* in mm/year) were estimated using the following equations.

6 $$GWE{T}_{i}=WBE{T}_{BC,i}$$

7 $$BWE{T}_{i}=EBE{T}_{BC,i}-WBE{T}_{BC,i}$$

To enable comparison of the two sources of ET over different landscapes, we derived percent ET source as shown below.

8 $$GWE{T}_{i}( \% )=\frac{WBE{T}_{BC,i}}{EBE{T}_{BC,i}}\times 100$$

9 $$BWE{T}_{i}( \% )=\frac{EBE{T}_{BC,i}-WBE{T}_{BC,i}}{EBE{T}_{BC,i}}\times 100$$

Finally, estimates of GWET and BWET were aggregated per land cover class or irrigated area boundary, which may be appropriate for regional planning purposes.

### Analysis of BWET and GWET for different land cover classes and irrigated areas

Previous research has focused on the variability in BWET over croplands and/or irrigated regions^6, 7, 13^. Our current understanding of the relative contribution and variability in GWET and BWET over other land cover types with respect to total ET is still limited. In this study, we use the MODIS 500-m land cover climatology product^18^ to summarize and understand GWET and BWET of 17 land cover types. The mean estimate of GWET and BWET for each land cover is summarized and presented for the period from 2001–2015. Since MODIS cropland class is a combination of rainfed and irrigated regions, we used irrigated area map obtained from the USGS^19^ for summarizing GWET and BWET contribution to total ET from irrigated areas.

ET requirement varies based on the geographic location and hydro-climatology, which influence water availability and atmospheric water demand. We summarized GWET and BWET estimates from 16 irrigated areas, spatially distributed from west to east. The location of these irrigated areas is provided in Fig. 7. The size of the selected irrigated areas varies from ~31,000 ha (Wilder Irrigation District, Idaho) to ~477,000 ha (Lower San Juaquin Irrigation District) with a mean area of 140,000 ha. These large areas of irrigation show high variability in irrigation intensity and crop type and thus, result in high spatial variability in BWET. To understand the temporal variability of BWET for each irrigated area, we analyzed magnitude of mean, ±1 standard deviation, and coefficient of variability of annual BWET estimates for each irrigation district. We also analyzed spatial variability in BWET within an irrigation district and compared the demand of BWET for different hydro-climates. All the data in this study is published online^39^.

## Electronic supplementary material

Supplementary Info
