# Supplementary material for: Partitioning Evapotranspiration into Green and Blue Water Sources in the Conterminous United States
Source: Sci Rep. 2017 Jul 21;7:6191. doi: 10.1038/s41598-017-06359-w (PMC5522464; doi:10.1038/s41598-017-06359-w)
Supplement: Supplementary file 1 — Supplementary Info [file 41598_2017_6359_MOESM1_ESM.pdf]

Partitioning Evapotranspiration into Green and Blue Water Sources in the Conterminous United States

Naga Manohar Velpuri[1*] and Gabriel B Senay[2]

[1]ASRC InuTeq LLC, Contractor to the U.S. Geological Survey (USGS) Earth Resources Observation and Science (EROS) Center, Sioux Falls, SD, USA

[2]U.S. Geological Survey (USGS), Earth Resources Observation and Science (EROS) Center, North Central Climate Science Center, Fort Collins, CO, USA

* communicating author (manohar.velpuri.ctr@usgs.gov)

Supplementary Information:

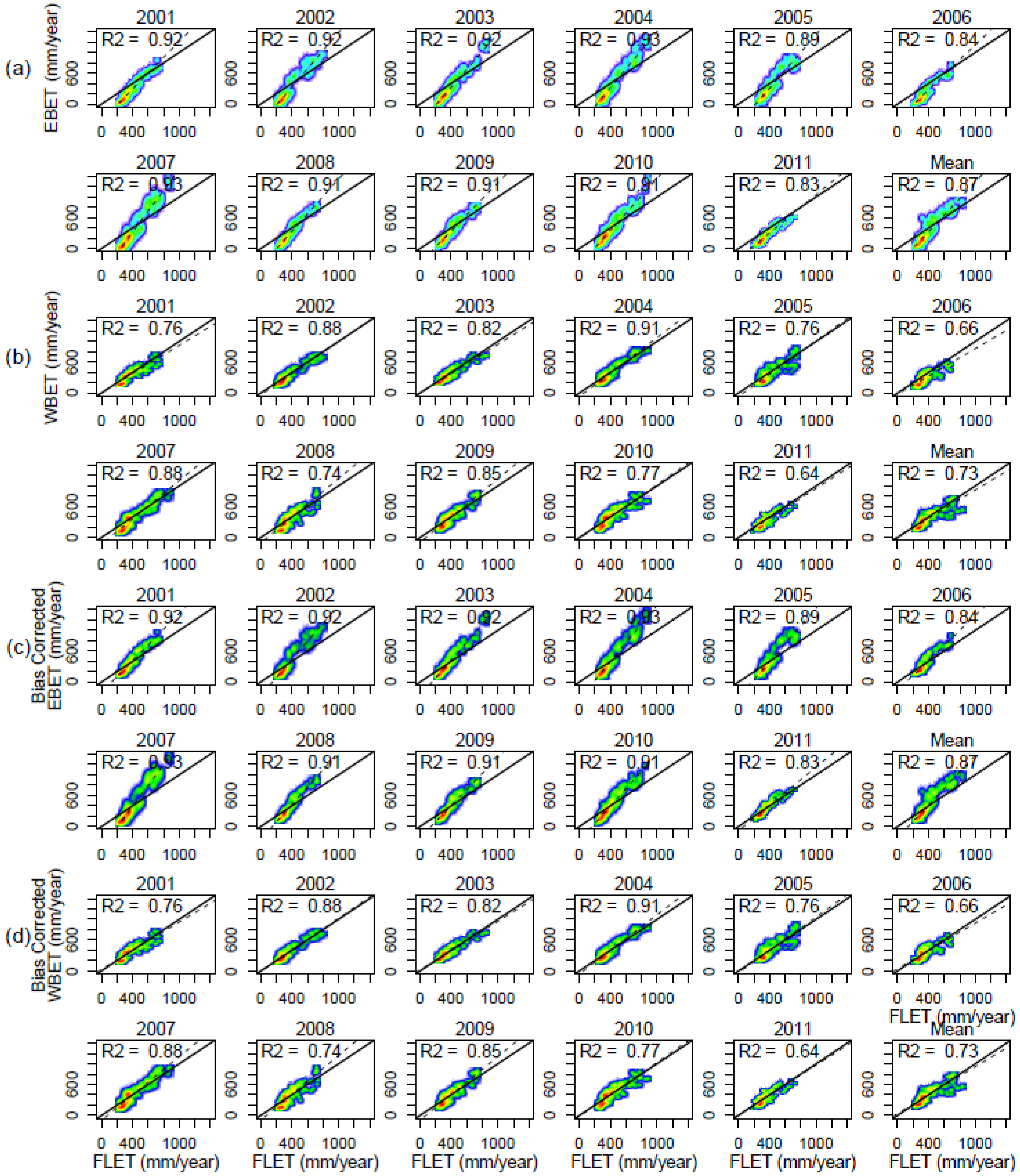

Figure 1. Scatterplots showing (a) energy balance ET vs MPI-ET, (b) water balance ET vs. MPI-ET, (c) Bias-corrected EBET vs. MPI-ET, and (d) bias-corrected WBET vs. MPI-ET.

Supplemental Table 1. Classification of selected irrigated areas into different hydro-climatic zones based on updated Koppen-Geiger climate classification map.

No	Irrigation District Name	Irrigation District Code	State	State Code	Area (Km^2)	Koppen-Geiger Climate Classification			
							Description		
						Code	Main Climate	Precipitation	Temperature
1	Palo Verde Irrigation District	PVID	California	CA	656	BWh	Arid	Desert	hot arid
2	Lower San Juaquin Irrigation District	LSJID	California	CA	4774	BSk	Arid	Steppe	cold arid
3	Glen-Colusa Irrigation District	GCID	California	CA	1721	Csa	Warm	summer dry	hot summer
4	Wilder Irrigation District	WID	Idaho	ID	309	BSk	Arid	Steppe	cold arid
5	Upper San Juaquin Irrigation District	USJID	California	CA	882	Csa	Warm Temperate	summer dry	hot summer
6	Las Cruces-Vado Irrigation	LCVI	New Mexico	NM	423	BWk	Arid	Desert	cold arid
7	Columbia Irrigation District	CID	Washington	WA	1510	Csb	Warm	summer dry	warm
8	Monte Vista Irrigation	MVID	Colorado	CO	2251	BSk/Dfb	Arid/Snow	Steppe/fully	cold
9	High Plains Irrig District	HPID	Texas	TX	1282	BSk	Arid	Steppe	cold arid
10	Nebraska Irrigation	NEI	Nebraska	NE	905	Dfa	Snow	fully humid	hot summer
11	Mason County Irrigation, IL	MCID	Illinois	IL	1492	Dfa	Snow	fully humid	hot summer
12	Mississippi-valley Irrigation	MID	Arkansas	AR	1192	Cfa	Warm	fully humid	hot summer
13	Flint River basin Irrigation	FID	Georgia	GA	650	Cfa	Warm	fully humid	hot summer
14	Florence Irrigation-SC	FISC	South Carolina	SC	1037	Cfa	Warm	fully humid	hot summer
15	Delaware River basin Irrigation	DRBI	Delaware	DE	1565	Cfa	Warm Temperate	fully humid	hot summer
16	Everglades Agriculture Area	EAA	Florida	FL	1970	Am	Equatorial	Monsoonal	-
